# Mott Cell-Featuring Inflammatory Bowel Disease with DNA Polymerase Epsilon Resolved by Hematopoietic Cell Transplantation

**DOI:** 10.1093/ibd/izaf224

**Published:** 2025-10-29

**Authors:** Ichiro Takeuchi, Chizuko Haga, Kentaro Fujimori, Toru Uchiyama, Toshinao Kawai, Kanako Tanase-Nakao, Ohsuke Migita, Ayame Ogawa, Akihiro Iguchi, Hirotaka Shimizu, Kosuke Taniguchi, Kimikazu Matsumoto, Kenichiro Hata, Hidenori Akutsu, Takashi Kato, Satoshi Narumi, Takako Yoshioka, Akira Ishiguro, Katsuhiro Arai

**Affiliations:** Center for Pediatric Inflammatory Bowel Disease, Division of Gastroenterology, National Center for Child Health and Development, Tokyo, 157-8535, Japan; Department of Pathology, National Center for Child Health and Development, Tokyo, Japan; Children Cancer’s Center, Division of Hematology, National Center for Child Health and Development, Tokyo, Japan; Department of Genome Medicine, National Research Institute for Child Health and Development, Tokyo, Japan; Division of Immunology, National Center for Child Health and Development, Tokyo, Japan; Department of Molecular Endocrinology, National Research Institute for Child Health and Development, Tokyo, Japan; Department of Maternal-Fetal Biology, National Research Institute for Child Health and Development, Tokyo, Japan; Department of Laboratory Medicine, St. Marianna University School of Medicine, Kanagawa, Japan; Department of Biology, Faculty of Education and Integrated Arts and Sciences, Waseda University, Tokyo, Japan; Integrative Bioscience and Biomedical Engineering, Graduate School of Advanced Science and Engineering, Waseda University, Tokyo, Japan; Children Cancer’s Center, Division of Hematology, National Center for Child Health and Development, Tokyo, Japan; Center for Pediatric Inflammatory Bowel Disease, Division of Gastroenterology, National Center for Child Health and Development, Tokyo, 157-8535, Japan; Department of Maternal-Fetal Biology, National Research Institute for Child Health and Development, Tokyo, Japan; Department of Human Molecular Genetics, Gunma University Graduate School of Medicine, Gunma, Japan; Children Cancer’s Center, Division of Hematology, National Center for Child Health and Development, Tokyo, Japan; Department of Maternal-Fetal Biology, National Research Institute for Child Health and Development, Tokyo, Japan; Department of Human Molecular Genetics, Gunma University Graduate School of Medicine, Gunma, Japan; Center for Regenerative Medicine, National Research Institute for Child Health and Development, Tokyo, Japan; Department of Biology, Faculty of Education and Integrated Arts and Sciences, Waseda University, Tokyo, Japan; Integrative Bioscience and Biomedical Engineering, Graduate School of Advanced Science and Engineering, Waseda University, Tokyo, Japan; Department of Pediatrics, Keio University School of Medicine, Tokyo, Japan; Department of Pathology, National Center for Child Health and Development, Tokyo, Japan; Clinical Resarch Center, National Center for Child Health and Development, Tokyo, Japan; Department of Pediatrics, National Hospital Organization Disaster Medical Center, Tokyo, Japan; Center for Pediatric Inflammatory Bowel Disease, Division of Gastroenterology, National Center for Child Health and Development, Tokyo, 157-8535, Japan

**Keywords:** congenital anemia, hematopoietic cell transplantation, inflammatory bowel disease, Mott cell, polymerase epsilon

Key Messages **What is already known?**

- Monogenic inflammatory bowel disease (IBD) can be caused by defects in immune regulation or epithelial integrity, but DNA polymerase–related mechanisms are rare and poorly understood.

**What is new here?**

- This is the first report of IBD associated with polymerase ε1 dysfunction featuring histopathological findings of Mott cells, with remission achieved through hematopoietic cell transplantation.

**How can this study help patient care?**

- Recognition of Mott cells in patients with IBD may prompt genetic evaluation for DNA polymerase dysfunction, leading to definitive treatment such as hematopoietic cell transplantation.

## Introduction

Inflammatory bowel disease (IBD) resulting from single gene abnormalities, termed “monogenic IBD,” has been linked to more than 100 genes to date.^1^ Immune cell dysregulation and epithelial barrier defects are the primary pathogenic mechanisms, whereas DNA polymerase dysfunction can also lead to enterocolitis. Among the major eukaryotic DNA polymerases (α, δ, and ε), *POLA1*, which encodes the catalytic subunit of DNA polymerase α, has been associated with monogenic IBD and X-linked reticulate pigmentary disorder (XLPDR).^2^ While patients with XLPDR reportedly develop early-onset colitis, enterocolitis attributable to dysfunction of other DNA polymerases has not been reported.

Previously, we identified pathogenic variants in *POLE* (p.[Asp1131fs];[Thr1891del]) (ClinVar accession numbers: SCV003806449 and SCV003806448, respectively), encoding polymerase ε1 (POLE1) in siblings presenting with severe congenital anemia.^3^^,^^4^ One sibling subsequently developed a unique form of infantile-onset IBD. We describe the endoscopic and histopathological characteristics of POLE1 dysfunction–associated IBD, providing novel insights into the potential role of POLE dysfunction in the pathogenesis of monogenic IBD.

## Methods

**Figure 1.**
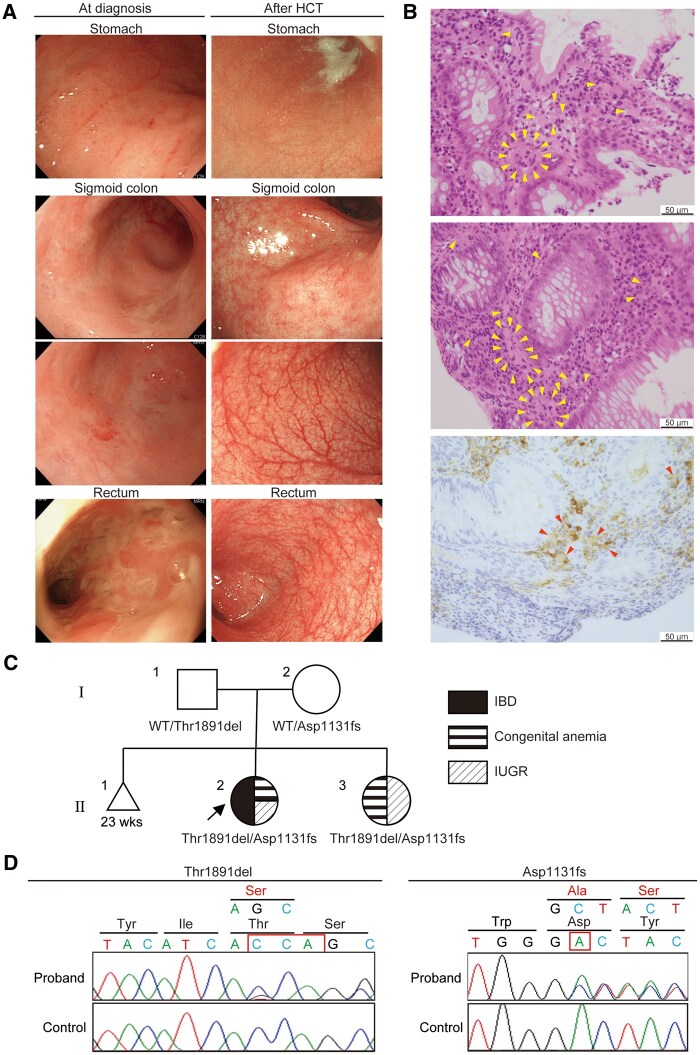
Clinical and genetic features in the pediatric patient with inflammatory bowel disease (IBD). (A) Endoscopic images obtained at diagnosis (left) and after hematopoietic cell transplantation (HCT) (right). Gastric erosions and geographic ulcers in the sigmoid colon and rectum resolved following HCT. (B) Histopathological examination of rectum at diagnosis, showing inflammatory lesions with Mott cells (yellow arrows). Immunohistochemical staining results reveal that Russell bodies in these cells are weakly IgA-positive (red arrows). (C) A pedigree chart. (D) Sanger sequencing results of the patient. IUGR, intrauterine growth restriction; WT, wild-type.

A Japanese girl was born at 29 weeks’ gestation with intrauterine growth restriction (IUGR) (weight: 726 ± −3.3 g and height of 32 ± −2.9 cm) and no significant family history. She required repeated red blood cell transfusions for severe congenital anemia. Bone marrow aspiration revealed no evidence of malignancy, myelodysplastic syndrome (MDS), or characteristic features of known congenital anemias (eg, Diamond-Blackfan anemia), consistent with our previous report. She developed diarrhea and bloody stool at 2 months of age. After excluding food protein–induced enterocolitis syndrome by introducing an amino acid–based hypoallergenic formula and infectious etiologies by stool culture, esophagogastroduodenoscopy and sigmoidoscopy at 6 months of age revealed geographic ulcers in the sigmoid colon and rectum and erosions in the stomach (Figure 1A). Histopathology did not show findings of cytomegalovirus infection but revealed chronic inflammatory changes characterized by infiltration of plasma cells and lymphocytes, along with architectural distortion such as twisted, branched, and angulated crypts. In addition, Mott cells were observed at both inflammatory sites in the stomach and rectosigmoid and noninflammatory sites of the esophagus and duodenum (Figure 1B). These Mott cells, characterized by Russell bodies, which are large eosinophilic intracytoplasmic inclusions, and eccentric nuclei, were confirmed as CD138-positive plasma cells. The Russell bodies were Igκ-positive, Igλ-negative, and weakly IgA-positive (Figure 1B). Ultrasonography showed no small intestinal wall thickening. Based on a diagnosis of IBD, 5-aminosalicylic acid and exclusive enteral nutrition were initiated; however, the disease was unresponsive. Ileocolonoscopy was deferred due to the high risk of perforation associated with severe inflammation and the patient’s small body size; however, sigmoidoscopy at 8 months of age, prior to the initiation of corticosteroid therapy, confirmed inflammation in the rectosigmoid region and Mott cells at the inflammatory sites. After the introduction of corticosteroids, the patient achieved remission. Azathioprine with parenteral nutrition was initiated but then discontinued because of lymphocytopenia, resulting in corticosteroid-dependent IBD. Although the flow cytometry results—including B cell counts—were not significant, her serum IgA levels were abnormally high (Table 1). Bone marrow aspiration at 17 months of age was again inconsistent with myelodysplastic features; however, aspiration at 22 months confirmed MDS. Consequently, hematopoietic cell transplantation (HCT) was performed to address her hematological disorder as previously reported.^3^ In addition to correcting her blood dyscrasia, HCT also resolved her IBD and high IgA levels (Table 1). Although she required a brief course of corticosteroids and infliximab for gut graft-versus-host disease that developed 6 months post-HCT, IBD remained in clinical remission for over 5 years without further medication. Endoscopic evaluation at 6 years of age revealed complete mucosal healing (Figure 1A), while histopathological analysis revealed no Mott cells. The patient exhibited mild developmental delay and facial dysmorphism with epicanthic folds. Despite adequate caloric intake post-HCT, her catch-up growth has remained suboptimal, although anemia or IBD did not relapse.

### Results

Whole-exome sequencing as a trio analysis was performed on fibroblasts derived from the patient’s skin, peripheral blood from her younger sister (II-3), and saliva from both parents to identify the underlying genetic cause of her complex phenotype. No causative genes of known monogenic IBD were found; however, pathogenic *POLE* variants (p.[Asp1131fs];[Thr1891del]), which encode POLE1, were identified. Our previous study demonstrated that these variants cause DNA POLE dysfunction, accompanied by p53 hyperactivation.^4^ The patient’s younger sister (II-3), who also had IUGR, had congenital anemia, and carried the same *POLE* variants, did not develop IBD before undergoing HCT for hematological issues, including progression from congenital anemia to MDS at 17 months of age.^3^^,^^4^

**Table 1.** Immunological characteristics of the patient with POLE1 dysfunction.

	7 mo of age (at diagnosis of IBD)	Reference range	11 months	Reference range	22 mo of age (before HSCT)	Reference range	28 mo of age (after HSCT)	Reference range
**Absolute lymphocyte count, cells/μL**	1200	3400-9000^a^			530	3600-8900^a^		
**Lymphocyte subsets**								
** CD3^+^ T cells/μL**	690	1900-5900^a^			889	2100-6200^a^		
** CD4^+^ T cells/μL**	570	1400-4300^a^			100	1300-3400^a^		
** CD25^+^CD127^+^ regulatory T cells/μL**	33				2			
** CD45RA^−^CXCR5^−^ (/μL)**	133							
** CD45RA^−^CXCR5^+^ (/μL)**	43							
** CD8^+^ T cells/μL**	90	500-1700^a^			130	620-2000^a^		
** CD19^+^ B cells/μL**	340	610-2600^a^			200	720-2600^a^		
** CD38^high^IgM^−^ plasmablasts/μL**	8				5			
** CD38^+^IgM^high^ transitional B cells/μL**	46				32			
** CD27^+^IgM^−^ class/switch recombination B cells/μL**								
** CD27^+^ memory B cells/μL**	68							
** CD16^+^CD56^+^ NK cells/μL**	110	160-950^a^			40	180-920^a^		
** CD56^+^CD3^+^ NKT cells/μL**	8				6			
**Immunoglobulin profile**								
** IgG level, mg/dL**	377	290-950^b^	513	380-1020^b^	950	380-1020^b^	487	500-1280^b^
** IgA level, mg/dL**	60	8-50^b^	306	10-59^b^	1090	10-59^b^	119	^b^18-123^b^
** IgM level, mg/dL**	46	46-176^b^	54	58-209^b^	47	58-209^b^	24	84-323^b^

Abbreviations: HSCT, hematopoietic stem cell transplantation; IBD, inflammatory bowel disease; NK, natural killer;

a Data quoted from Shear WT, Rosenblatt HM, Gelman RS. Lymphocyte subsets in healthy children from birth to 18 years of age: the Pediatric AIDS Clinical Trials Group P1009 study. *J Allergy Clin Immunol.* 2003;112:973-980.

b Data quoted from the reference intervals of clinical tests in Japanese children, from Tanaka T, Yamashita A, Ichihara K. Reference intervals of clinical tests in children determined by a latent reference value extraction method. *J Jpn Pediatr Soc.* 2008;112:1117-1132.

This study was approved by the Institutional Review Board of the National Center for Child Health and Development, Tokyo, Japan (#378, #926). Written informed consent was obtained from the patient’s parents.

## Discussion

This is the first report to describe IBD associated with POLE1 dysfunction, involving both the colon and upper gastrointestinal tract, as well as the distinctive presence of Mott cells in histopathology. Although IBD was initially dependent on corticosteroids, HCT proved effective in achieving remission. *POLE* has previously been implicated in FILS (facial dysmorphism, immunodeficiency, livedo, short stature) and IMAGE-I (IUGR, metaphyseal dysplasia, adrenal hypoplasia congenita, genital anomalies, and immunodeficiency) syndromes.^5^^,^^6^ However, no associations with IBD have been reported to date. We previously demonstrated that the POLE1 protein encoded by *POLE* (p.[Asp1131fs];[Thr1891del]) fails to translocate into the nucleus and function as a DNA polymerase, causing p53 overexpression.^4^ We hypothesize that these variants help explain the presence of congenital anemia in our cases, in contrast to FILS and IMAGE-I syndromes, in which at least 1 of the pathogenic variants lies within an intronic region (c.4444 + 3A>G in FILS and c.1226 + 234G>A or c.1686 + 32C>G in IMAGE-I) leaking residual expression of functional POLE1, although a quantitative genotype-severity correlation remains to be established.^5^^,^^6^ While IBD has not been reported in these syndromes, the more severe POLE1 dysfunction and heightened DNA stress observed in our patient may have contributed to the development of IBD. Because DNA stress can trigger chronic inflammation through interferon production, the exceptional *POLE* mutation–induced DNA stress in our patient may have driven chronic inflammation via interferon-related pathways.^7^

This case of IBD associated with POLE1 dysfunction, along with the histopathological findings of Mott cells, may represent the underlying pathogenesis. Mott cells are a type of plasma cells that produce large amounts of immunoglobulins forming eosinophilic inclusions called Russell bodies, and are commonly observed in multiple myeloma or autoimmune disorders during exaggerated inflammatory responses.^8^ Their presence suggests aberrant plasma cell activity and chronic immune stimulation. In XLPDR, another DNA polymerase–related disease, polymerase dysfunction leads to aberrations in DNA and RNA species, which promote type I interferon production.^9^ Type I interferons are pivotal cytokines in autoimmune diseases, facilitating B cell differentiation into autoantibody-secreting plasma cells.^10^ While interferon levels were not measured in our patient, and the etiology of IgA hyperproduction remains uncertain, the presence of IgA-positive Russell bodies within Mott cells, together with elevated serum IgA levels, points toward plasma cell hyperactivation as a possible mechanism underlying chronic inflammation.^8^ The treatment responses of IBD to corticosteroids and the resolution by HCT, which can recalibrate immune responses, further supports a pathogenic process driven by excessive plasma cell activity potentially triggered by POLE1 dysfunction.

Directly validating a causal link between POLE1 dysfunction and IBD remains challenging due to lethality in animal models, and the unaffected status of the patient’s sibling with the same variants remains unexplained. Even monogenic IBD can be influenced by interactions among genetic factors, immune responses, and gut microbiota, exemplified by the incomplete penetrance observed in cytotoxic T lymphocyte–associated protein 4 or X-linked inhibitor of apoptosis protein deficiency, both of which are recognized causes of monogenic IBD.^1^ Consequently, additional triggers beyond POLE1 dysfunction may be necessary for IBD manifestation.

Although this study presents only a single case, our findings broaden the clinical phenotypes attributed to POLE1 dysfunction and highlight its potential roles in IBD development. Genetic testing for polymerase dysfunction should be considered in patients whose histopathological analysis reveals the presence of Mott cells, particularly when accompanied by features overlapping with FILS or IMAGE-I syndromes and/or hematological abnormalities. Furthermore, this case highlights the underrecognized roles of defects in DNA synthesis in the pathogenesis of chronic inflammatory conditions.

## Data Availability

The data on which the conclusions of this study are based are available from the corresponding author upon reasonable request.
